# A commentary on ‘The obesity challenge in joint replacement: a multifaceted analysis of self-reported health status and exercise capacity using NHANES data - a population-based study’

**DOI:** 10.1097/JS9.0000000000001523

**Published:** 2024-05-28

**Authors:** Mengxian Wei, Yuquan Chen, Yanwei You, Xindong Ma

**Affiliations:** a Division of Sports Science and Physical Education, Tsinghua University; b School of Social Sciences, Tsinghua University; c IDG/McGovern Institute for Brain Research, Tsinghua University, Beijing, People’s Republic of China; d School of Public Health and Preventive Medicine, Faculty of Medicine, Nursing and Health Sciences, Monash University, Melbourne, Victoria, Australia

*Dear Editor,*

We read with great interest the recent publication titled ‘The obesity challenge in joint replacement: a multifaceted analysis of self-reported health status and exercise capacity using NHANES data - a population-based study’ in the *International Journal of Surgery* ^1^. This study provides important insights into the complex relationship between obesity, self-reported health status, and exercise capacity among joint replacement patients, using data from the National Health and Nutrition Examination Survey (NHANES). The study’s findings, particularly the observation that severely obese patients did not exhibit a significantly elevated risk of poor/fair self-reported health compared to normal-weight subjects, are both intriguing and clinically relevant. Furthermore, the identification of physical functioning as a robust predictor of self-reported health underscores the importance of considering individual physical abilities in the treatment of joint replacement patients. Although the study presents valuable insights into the obesity status in joint replacement using the NHANES database, it is important to acknowledge several limitations that may affect the interpretation of the results (as summarized in Fig. 1).

**Figure 1:**
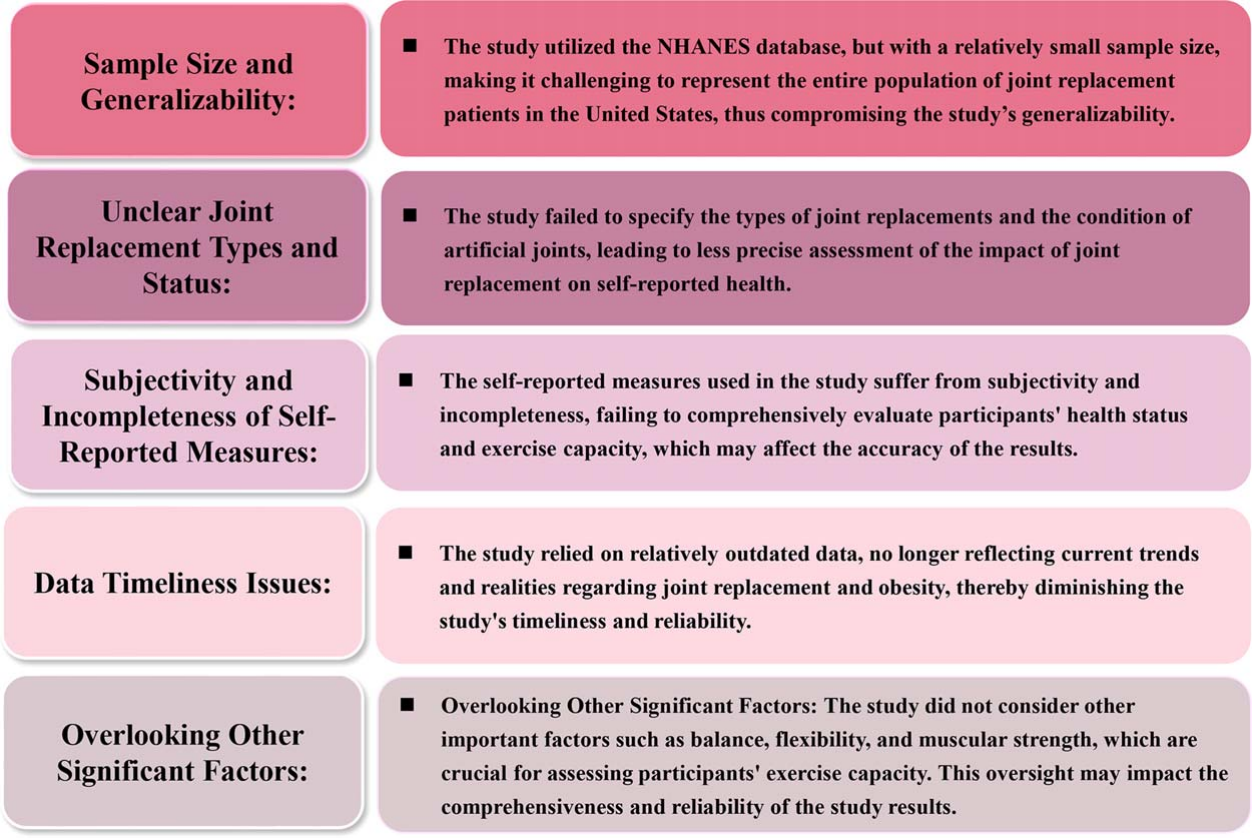
The obesity challenge in joint replacement: potential limitations.

Firstly, the study’s reliance on NHANES data, while providing a comprehensive view of the U.S. population’s health status, involves certain limitations when extrapolating findings from a sample of over 300 individuals to represent over 3 million. Despite employing robust statistical methods for data weighting and stratification, the inherent variability and potential biases within the larger population may not be fully captured^2^. Therefore, readers should interpret the study’s findings with caution, considering the potential limitations in generalizability.

Secondly, the inability to determine specific joint replacements, such as hip or knee replacements, and the state of the artificial joint at the time of the survey poses challenges in accurately assessing the impact of joint replacement on self-reported health status. Additionally, the selection of patients with functional limitations may skew the sample toward individuals with more severe disease, which could affect the study’s findings. Moreover, the lack of information regarding the duration of arthroplasty surgery and the potential progression of prosthetic wear further complicates the interpretation of the results. While the study acknowledges these limitations, their specific impact on the study’s outcomes remains unclear.

Thirdly, the study relies on self-reported measures to assess both health status and functional outcomes. Using a Likert-like scale for self-reported health status and limited assessment of functional outcomes may introduce subjectivity, response bias, and an incomplete understanding of participants’ overall health and exercise capacity. The dichotomization of responses into poor or fair health versus good, very good, or excellent health oversimplifies the nuanced nature of health perceptions and may overlook important distinctions within these categories. Additionally, incorporating validated assessment tools, objective measures, and a comprehensive evaluation of functional outcomes could enhance the rigor and accuracy of the study’s results, providing a more nuanced understanding of the relationship between obesity, self-reported health, and exercise capacity among joint replacement patients^3^. Other important aspects of exercise capacity, such as balance, flexibility, and muscular strength, are not assessed. Using validated functional assessment scales such as the Short Physical Performance Battery (SPPB), Timed Up and Go Test (TUG), 6-Minute Walk Test (6MWT), etc., enables a more comprehensive evaluation of participants’ exercise capacity^4^. Moreover, objective measures, such as accelerometer data and muscle quality index assessment conducted by healthcare professionals^5^, could provide more reliable and valid data on participants’ health status and functional abilities.

In addition to the mentioned limitations, another notable drawback of the study is the utilization of relatively outdated data. The study participants were assessed using data from three cycles spanning from 1 January 1999 to 31 December 2004. Given the rapidly evolving nature of healthcare practices, technological advancements, and changes in population demographics over time, relying on data that is over two decades old may not accurately reflect the current trends and realities regarding joint replacement and obesity.

In conclusion, while the study offers valuable insights into the relationship between obesity and joint replacement, readers should consider the aforementioned limitations when interpreting the results. Future research with larger, more direct sample sizes and detailed clinical data could provide further validation and enhance our understanding of the complex relationship between obesity and health status among joint replacement patients.

## Ethical approval

Not applicable.

## Consent

Not applicable.

## Source of funding

This study was supported by the Institute of Sports Development Research of Tsinghua University (Research on John Mo’s thought and practice of Physical Education).

## Author contribution

M.W., Y.C., and Y.Y.: writing and figure format; M.W., Y.C., Y.Y., and X.M.: concept.

## Conflicts of interest disclosure

The authors declare that there are no conflicts of interest regarding the publication of this paper.

## Research registration unique identifying number (UIN)

Not applicable.

## Guarantor

Xindong Ma.

## Data availability statement

The data used to support the findings of this study are included within the article.

## Provenance and peer review

Not applicable.
